# Colposcopic Findings and Diagnosis in Low-Income Brazilian Women with ASC-H pap Smear Results

**DOI:** 10.1055/s-0042-1742289

**Published:** 2022-02-25

**Authors:** Cibele Feroldi Maffini, Luiz Martins Collaço, Ana Paula Martins Sebastião, Rita Maira Zanine

**Affiliations:** 1 Department of Obstetrics and Gynecology, Clinics Hospital of the University of Paraná, Curitiba, PR, Brazil; 2 Department of Pathology of Clinics Hospital of the University of Paraná, Curitiba, PR, Brazil; 3 Department of Gynecology and Obstetrics, Lower Genital Tract Disease and Colposcopy Sector, Clinics Hospital of the University of Paraná, Curitiba, PR, Brazil

**Keywords:** colposcopy, cervical intraepithelial neoplasia, cervical cancer, ASC-H, cervix uteri, colposcopia, neoplasia intraepithelial cervical, câncer de colo uterino, células escamosas atípicas do colo do útero, colo do útero

## Abstract

**Objective** To determine the accuracy of colposcopy findings in diagnosing cervical intraepithelial neoplasia (CIN) in women with an atypical squamous cells, cannot exclude high-grade squamous intraepithelial lesion (ASC-H) pap smear result and analyze whether the prevalence of HSIL and cancer correlates with sociodemographic risk factors and specific colposcopic findings.

**Methods** Colposcopic findings and sociodemographic risk factors were analyzed as possible predictors of a CIN 2 or worse diagnosis in women with an ASC-H pap smear result.

**Results** Accuracy of the colposcopic impression was 92%, sensitivity was 91.6%, and specificity was 93.1%, with a positive predictive value of 96.4% and negative predictive value of 84.3%. Diagnosis of CIN 2 or worse was more frequent in patients with a previous history of cervical dysplasia and pre-menopausal patients. Identification of major colposcopic findings, dense acetowhite epithelium, coarse mosaicism, and punctuation correlated significantly with CIN 2 or worse.

**Conclusion** Colposcopy performed by an experienced examiner can accurately differentiate patients with CIN 1 or less from patients with CIN 2 or worse. Diagnosis of CIN 2 or worse was more frequent in patients with a previous history of cervical dysplasia and pre-menopausal patients. The degree of acetowhite changes was the best colposcopic feature to predict CIN2 or worse.

## Resumo

**Objetivo:** Determinar a acurácia dos achados colposcópicos no diagnóstico das neoplasias intraepiteliais cervicais (NIC) em mulheres com resultado de exame citopatológico de células escamosas atípicas de significado indeterminado não podendo excluir lesão intraepitelial de alto grau (ASC-H) e analisar a correlação entre a prevalência de HSIL ou câncer com fatores de risco sociodemográficos e achados colposcópicos específicos.

**Métodos:** Os achados colposcópicos, e os fatores de risco sociodemográficos foram analisados como possíveis preditores de diagnóstico NIC 2 ou mais grave em mulheres com resultado de exame citopatológico ASC-H.

**Resultados:** A acurácia da impressão colposcópica foi de 92%, sensibilidade foi 91,6%, e a especificidade foi de 93,1%, com um valor preditivo de 96,4% e valor preditivo negativo de 84,3%. O diagnóstico de NIC 2 ou mais grave foi mais frequente em pacientes com história pregressa de displasia cervical e nas que não estavam na pós menopausa. A identificação de achados colposcópicos maiores, epitélio acetobranco denso, mosaico e pontilhados grosseiros se correlacionaram positivamente com o diagnóstico NIC 2 ou mais grave.

**Conclusão:** A colposcopia realizada por um examinador experiente pode diferenciar com acurácia pacientes com NIC 1 ou menos grave de pacientes com NIC 2 ou mais grave. O diagnóstico de NIC 2 ou mais grave foi mais frequente em pacientes com história pregressa de displasia cervical e pacientes que estavam na pré menopausa. A densidade da acetorreação foi o melhor preditor colposcópico para NIC 2 ou mais grave.

## Introduction

The cytological diagnosis of atypical squamous cells, cannot exclude high-grade squamous intraepithelial lesion (ASC-H) is a relatively new cytological classification for atypical squamous cells formally introduced with the 2001 Bethesda system. 1 Because of the high incidence of clinically significant lesions noted on subsequent follow-up, it has been suggested that patients with an ASC-H pap smear result be observed closely and referred for colposcopic examination. 2 3

The management of a cervical cytological lesion depends on the severity of the lesion and its inherent underlying or future risk of high-grade cervical intraepithelial neoplasia (CIN) or cancer. 3 A low-grade squamous intraepithelial lesion (LSIL) often regresses spontaneously, especially in young women, whereas a high-grade squamous intraepithelial lesion (HSIL) is more likely to persist or even progress to more severe lesions. 4 5 Therefore, HSIL diagnosis and treatment is the main goal of cervical cancer screening and prevention programs. The aim of the colposcopic examination is to correctly identify patients at high risk of developing cervical cancer and refer them for excisional or destructive treatment. Interpreting colposcopic epithelial patterns and subsequently selecting the site for biopsy is a subjective procedure that correlates strongly with the skill and experience of the colposcopist. 6 The number of biopsies, type of human papilloma virus (HPV), the size of the lesion, and its severity are potential factors that may influence the accuracy of colposcopy. 7 8 9 The sensitivity of colposcopy ranges from 64 to 99% and the specificity from 30 to 93%. 10

This study aimed to investigate the diagnostic performance of colposcopy performed by a highly experienced examiner using the International Federation for Cervical Pathology and Colposcopy (IFCPC) nomenclature in a low-income Brazilian population with an ASC-H pap smear result. 11 This study also aimed to analyze whether the prevalence of HSIL and cancer correlates with sociodemographic risk factors and specific colposcopic findings. We believe this knowledge can potentially help develop clinical protocols for selecting cases for a “see and treat” approach in the ASC-H cytological category.

## Methods

This study was conducted in the city of Curitiba at the Clinics Hospital of the University of Paraná, a Brazilian institution responsible for patients requiring a higher level of health care.

Between July 2009 and August 2016, all women with cytological diagnosis of atypical squamous cells, cannot exclude high-grade squamous intraepithelial lesion (ASC-H) or high-grade squamous intraepithelial lesion (HSIL) referred to the Lower Genital Tract Disease and Colposcopy specialized service, were analyzed.

Colposcopic examinations performed by the same highly experienced colposcopist (with 30 years of experience performing over 4,000 colposcopies/year) were selected. Biopsies were taken from the area with the worst colposcopic impression according to the standard practice.

After approval from the local ethical committee colposcopic findings were collected from medical records and colposcopic impressions were classified according to the International Federation for Cervical Pathology and Colposcopy (IFCPC) criteria as either negative, Grade 1 (minor), or Grade 2 (major). 11

The sociodemographic risk factors for cervical cancer collected from medical records were age, parity, menopausal status, lifetime number of sexual partners, tobacco smoking, co-infection with HIV or other sexually transmitted infection, and previous medical history of cervical dysplasia.

The same staff pathologist blindly reviewed all cytology results using the same criteria used in daily practice. The reviewed cases were classified in accordance with the 2001 Bethesda System and the Brazilian Nomenclature for Cytopathological Reports. 12 Blinded to the previous cytology results, a second staff pathologist reviewed the remaining discordant cases. The final diagnosis was then defined by agreement with either the original diagnosis or the first staff pathologist, resulting in the diagnosis of ASC-H cases or HSIL cases. When agreement could not be reached the most severe diagnosis was used.

Histological diagnosis of a biopsy obtained by large loop excision of the transformation zone (LLETZ) or cold knife conization was considered the gold standard for diagnosis of CIN 2 or worse.

A diagnosis of CIN 1 or less was defined as a negative colpocytological result 6 and 12 months after the initial colposcopy. When a negative result was not achieved at follow-up, and a second abnormal pap smear result was obtained, the patient was then referred for LLETZ or cold knife conization and this histological diagnosis was considered the “gold standard”.

The inclusion criteria were: 1) aged 21 years and older, 2) not pregnant, 3) no recent treatment for cervical intraepithelial neoplasia, 4) no history of total hysterectomy, and 5) no history of radiotherapy treatment for invasive cervical carcinoma. The exclusion criteria were 1) colposcopic examination at another clinic, 2) no records of colposcopic examination, 3) inadequate colposcopy, 4) slides inadequate for cytology review panel.

We examined the accuracy of colposcopy findings for the prediction of two main diagnostic endpoints, CIN 1 or less (low risk of developing cervical cancer) and CIN 2 or worse (high risk of developing cervical cancer) in women with an ASC-H pap smear result.

Data were analyzed using IBM SPSS Statistics for Windows, Version 20.0 software (IBM Corp., Armonk, NY, USA). Results are presented as means, standard deviations, medians (with ranges), numbers, or frequencies, as appropriate. A Chi-squared or Fisher exact test was used to univariately identify factors related to the presence of the diagnostic endpoints. A multivariate analysis of factors predicting these endpoints was estimated using binary logistic regression featuring Hosmer-Lemeshow goodness-of-fit testing. All *p* -values ˂ 0.05 were considered statistically significant.

## Results

**Fig. 1:**
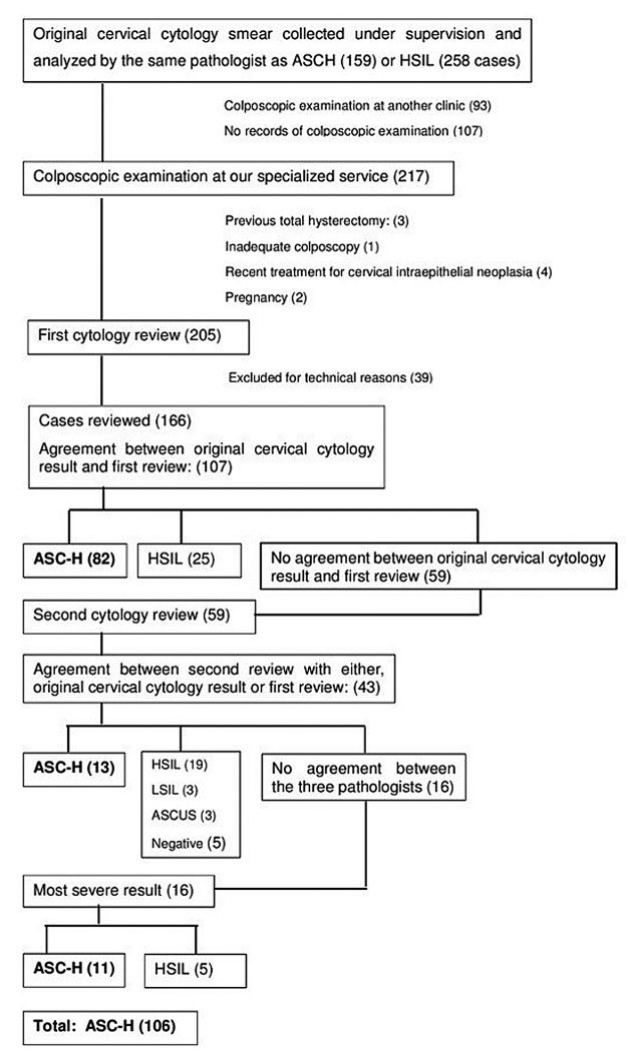
Case selection and peer review of cervical smears.

During the study period, 18,046 women were screened for cervical neoplasia with conventional cytology. Abnormal cervical cytology prevalence was 14.6%, of which 159 women with an ASC-H pap smear result and 258 with a HSIL finding were referred to the lower genital tract disease and colposcopy specialized service. After cytological panel review was complete 106 cases with an ASC-H result were selected for further analysis, *kappa* values for interobserver agreement between pathologists was 0,49 (moderate agreement) ( Fig. 1 ).

In terms of sociodemographic characteristics, the study population showed a high prevalence of tobacco smoking, HIV infection, and a previous history of HSIL treatment ( Table 1 ).

**Table 1:** Demographic and clinical characteristics of the study population

Characteristic	n	(%)
Age		
Median	36	N/A
Range	21 to 84	N/A
Lifetime number of sexual partners		
Median	2	N/A
Range	1 to 30	N/A
Menopause		
Yes	23	(21.7%)
No	83	(78.3%)
Tobacco smoking		
Yes	36	(34.0%)
No	70	(66.0%)
Coinfection with others sexually transmitted agents and HIV		
Yes	17	(16.0%)
No	85	(80.2%)
HIV	4	(38%)
Previous history of HSIL		
Yes	21	(19.8%)
No	85	(80.2%)

Abbreviations: HIV, human immunodeficiency virus; HSIL, high-grade squamous intraepithelial lesion.

After initial assessment, 65 patients with major colposcopic impressions were identified. In 60 cases, the histological assessment was compatible with HSIL or cancer. A total of 19 patients with major colposcopic impressions were referred to LLETZ without prior histologic diagnosis (see and treat” approach). In 18 cases, histological assessment of the LLETZ specimens confirmed the presence of HSIL and one patient was lost to follow-up before undergoing LLETZ treatment ( Table 2 ).

**Table 2:** Colposcopic impression and clinical and histopathologic outcome

	CIN 1 or less			CIN 2 or worse		Non-attendance	*P* -value ┼
Colposcopic Impression/Transformation Zone	Negative Clinical follow-up	LLETZ histopathologic results					
		Negative	CIN 1	CIN2/3	Cancer		
Major (grade 2) (n = 65)	0 (0.0)	2 (3.1)	2 (3.1)	53 (82.2)	7 (10.9)	1	< 0.001 *
TZ1 (n = 36)	0 (0.0)	1 (2.8)	1 (2.8)	31 (88.6)	2 (5.7)	1	
TZ2 (n = 14)	0 (0.0)	1 (7.1)	0 (0.0)	12 (85.7)	1 (7.1)	0	
TZ3 (n = 15)	0 (0.0)	0 (0.0)	1 (6.6)	10 (66.6)	4 (26.6)	0	
Minor (Grade 1) (n = 12)	6 (54.5)	0 (0.0)	3 (27.3)	2 (18.2)	0 (0.0)	1	< 0.001 *
TZ1 (n = 9)	5 (62.5)	0 (0.0)	2 (25.0)	1 (12.5)	0 (0.0)	1	
TZ2 (n = 1)	0 (0.0)	0 (0.0)	0 (0.0)	1 (100.0)	0 (0.0)	0	
TZ3 (n = 2)	1 (50.0)	0 (0.0)	1 (50.0)	0 (0.0)	0 (0.0)	0	
Negative (n = 29)	18 (66.7)	4 (14.8)	2 (7.4)	2 (7.4)	1 (3.7)	2	
TZ1 (n = 6)	6 (100.0)	0 (0.0)	0 (0.0)	0 (0.0)	0 (0.0)	0	
TZ2 (n = 4)	2 (66.6)	0 (0.0)	1 (33.3)	0 (0.0)	0 (0.0)	1	
TZ3 (n = 29)	10 (55.5)	4 (22.2)	1 (5.5)	2 (11.1)	1 (5.5)	1	
Total (n = 106)	24 (23.5)	6 (5.9)	7 (6.9)	57 (55.9)	8 (7.8)	4	

Abbreviations: CIN, cervical intraepithelial neoplasia; LLETZ, large loop excision of the transformation zone; TZ, transformation zone.

Data are presented as number (percentage) unless stated otherwise.

Non-attendance was not included in the calculation of proportions of histological results.

┼ comparing the incidence of CIN2/3and cancer between the groups with minor or negative versus major colposcopic impression. n, number of women in each category.

* statistically significant.

Twelve patients had minor colposcopic impressions, and only two (18.2%) had a final diagnosis of CIN 2 or worse. One was lost to follow-up. Negative colposcopic findings occurred in 29 cases and three (10.3%) of them had a final diagnosis of CIN 2 or worse. All of these cases had a type 3 transformation zone. Two cases were lost to follow-up ( Table 2 ).

A CIN 1 or less endpoint occurred in 37 (34.9%) patients and a CIN 2 or worse endpoint occurred in 65 (61.3%) patients. The non-attendance rate was 3.7%. Excluding non-attenders, the rate of HSIL diagnosis was 55.9% and the rate of invasive carcinoma diagnosis was 7.8%.

In this study, the sensitivity, specificity, and the positive and negative predictive values of colposcopy were determined to be 91.6% (95% CI, 81.6–97.2), 93.1% (95% CI, 77.2–99.1), 96.4% (95% CI, 87.8–99.0), and 84.3% (95% CI, 69.9–92.6), respectively. The overall accuracy of colposcopic impressions was 92%.

We applied the chi-square test in order to establish the correlation between the colposcopic impression result and the two main diagnostic endpoints. The colposcopic impression was statistically relevant with a p-value of < 0.001 for the detection of CIN 1 or less and CIN 2 or worse ( Table 2 ).

Univariate analysis showed a statistical correlation between all major findings and the CIN 2 or worse endpoint, and between minor findings and the CIN 1 or less endpoint ( Table 3 ). Interestingly, a transformation zone (TZ) type 3 finding seemed to be a statistically significant protective factor, whereas a smaller lesion size was not ( Table 3 ).

**Table 3:** Univariate analysis of colposcopic findings and main endpoints

Colposcopic findings	ENDPOINTS		*P* -value	Odds ratio
	CIN 1 or less	CIN 2 or worse		
Transformation zone				
Types 1 and 2	18 (48.6%)	48 (73.8%)	0.011 *	2.98 (1.27–6.96)
Type 3	19 (51.4%)	17 (26.2%)		
Number of cervical quadrants covered by the lesion				
0, 1, and 2	25 (67.5%)	49 (75.3%)	0.396	0.68 (0.27–1.65)
3 and 4	12 (32.4%)	16 (24.6%)		
Acetowhite changes				
Fine and absent	33 (89.2%)	8 (12.3%)	< 0.001 *	58.78 (16.5–210.2)
Dense	4 (10.8%)	57 (87.7%)		
Punctation				
Dense	1 (2.7%)	22 (33.8%)	0.005 *	18.42 (2.36–143.4)
Fine and absent	36 (97.3%)	43 (66.1%)		
Mosaicism				
Dense	1 (2.7%)	18 (28.1%)	0.011 *	14.08 (1.79–110.5)
Fine and absent	36 (97.3%)	46 (71.9%)		

* Statistically significant; CIN, cervical intraepithelial neoplasia.

Multivariate analysis showed that once dense acetowhite changes were present, there was a 63.7% odds ratio in favor of a CIN 2 or worse endpoint compared to CIN 1 or less. Although not statistically significant, only pre-menopausal status and a previous history of HSIL showed a tendency towards prediction of the two diagnostic endpoints ( Table 4 ).

**Table 4:** Univariate analysis of risk factors

	ENDPOINTS				*P* -value
	CIN 1 or less		CIN 2 or worse		
Menopause					
No	25	67.6%	54	83.1%	0.072
Yes	12	32.4%	11	16.9%	
Tobacco smoking					
No	27	73.0%	41	63.1%	0.308
Yes	10	27.0%	24	36.9%	
Coinfection with other sexually transmitted agents and HIV					
				
No	31	83.8%	52	80.0%	0.999
Yes	5	13.5%	10	15.4%	
HIV	1	2.7%	3	4.6%	
Previous history of HSIL					
No	26	70.3%	55	84.6%	0.085
Yes	11	29.7%	10	15.4%	
Age					
Average	41.54	SD 13.34	37.06	SD 12.42	0.091
Minimal	21		21		
First quartile	32		28		
Median	40		34		
Third quartile	50		43		
Maximal	74		84		
Lifetime sexual partners					
Average	3.92	SD 4.62	2.42	SD 2.42	0.817
Minimal	1		1		
First quartile	2		3		
Median	2		2.5		
Third quartile	3		4		
Maximal	20		10		

## Discussion

Given the higher complexity of the cases attending our hospital, it was not surprising that the rate of ASC-H in our laboratory was 0.88%, which is much higher than the average of 0.2% previously reported. 2

A pap smear result indicating atypical squamous cells that cannot exclude HSIL (ASC-H) may reflect a mixture of true HSIL and its mimics. These definitions leave some room for individual interpretation, as shown by Confortini et al., 13 who reported the lowest degree of agreement for the ASC-H category (specific *k* = 0.38) after a peer review of 63,754 smears. Therefore, the panel review of cytology results in this study aimed to reduce selection bias, what we consider a strength in our study. Interobserver agreement between pathologists in our study was considered moderate ( *k =* 0.49), similar to previously reported by Confortini et al. 13

In our study population, 63.7% of patients had a histologic diagnosis of CIN 2 or worse. Some authors have reported that approximately half (32–90%) of women with an ASC-H cytology result will have less than a HSIL diagnosis on their cervical biopsy sample. 14 15 Recently, a meta-analysis pooled over 4,000 ASC-H cases and estimated the prevalence of histopathological diagnosis of CIN 2 or worse to be 34% (CI 95%, 28–40) with a range of 13–66%. 16 The literature generally reports that the incidence of invasive disease in patients with an ASC-H result is rare (range 1–3%). 17 18 Our invasive carcinoma incidence (7.8%) was similar to the study of Nogara et al. 19 that reported a 7.9% incidence of invasive carcinoma. A higher prevalence of invasive carcinoma in an ASC-H population, such as the 9.3% prevalence of invasive carcinoma found in a study that only included patients with histological samples, is usually associated with different selection criteria. 20 The relatively high incidence of invasive disease in our study supported our classification of this population as “high-risk”.

Future analysis is essential to estimate the benefits of a “see and treat” approach in an ASC-H population. In our study, a “see and treat” approach was applied in 18 selected cases and overtreatment did not occur. This practice is an attractive option, as clinical and laboratory resources would be used more efficiently, and the possibility of non-compliance with follow-up visits for further treatment is also avoided. However, we cannot ignore the issue of overtreatment or potential morbidity of LLETZ in patients who may not require treatment. 21

The high incidence of invasive disease was probably a consequence of the high prevalence of sociodemographic risk factors. The proportion of tobacco smokers was 34% in our study population, whilst the national data shows that only about 15% of Brazilian women are regular smokers. 22 Another risk factor was the higher prevalence of seropositivity for HIV, found in 3.8% of patients, which is much higher than the 0.4 to 0.7% estimated by population-based data in Brazil. 23 There is no population data available on the prevalence of previous history of HSIL treatment in Brazil, but we considered 19.8% higher than expected. The mean age at the time of ASC-H cytology diagnosis was 38.7 years (median: 36 years), and this was similar to that previously reported by other studies such as Bonvicino et al. 24 (35.6 years) and Nogara et al. 19 (32.4 years).

Louro et al. 17 stated that patients 40 years or older were less likely to have a clinically significant lesion detected on subsequent histologic follow-up than patients younger than 40 years. In our findings, patients with a CIN 2 or worse endpoint were older than those presenting with CIN 1 or less. Although estrogen deficiency may lead to cytological changes that mimic HSIL, previous studies involving age group analyses have shown a higher prevalence of pre-invasive lesions in younger populations. 17 25 Univariate analysis of sociodemographic risk factors showed a borderline correlation between being pre-menopausal and a CIN 2 or worse endpoint with a *p* -value of 0.072. There is some evidence that the prevalence of CIN 2 or worse in ASC-H populations is lower after menopause. 17 25 Almost 20% (n = 21) of the study population reported a previous history of HSIL treatment 2 years or more before ASC-H cytology and metachronous lesions occurred in 11 (47.6%) cases. Several studies have demonstrated that women who had a previous diagnosis of a high-grade cervical lesion or invasive cervical cancer had an increased risk of developing metachronous lesions within the lower genital tract compared to women in the general population. 26 27 Despite this, our findings showed only a trend ( *p* = 0.085) towards a relationship between a previous history of HSIL and a CIN 2 or worse endpoint.

The borderline correlations in our study analysis may be the consequence of our limited sample size. Perhaps, in larger populations, these sociodemographic factors would have been more representative as risk factors. A TZ type 3 finding behaved as a protective factor in our study population, and this was probably a consequence of its frequent association with older and postmenopausal patients. In those cases, the ASC-H result was most likely associated with atrophy changes rather than CIN 2 or worse lesions.

The colposcopic impression findings were statistically relevant in the detection of the two diagnoses of CIN 1 or less and CIN 2 or worse. Its sensitivity, specificity, and positive and negative predictive values showed an expressively high performance, close to the high confidence interval margins previously reported. 10 28 29

The chi-square test showed that grade 2 (major) colposcopic impression findings correlated significantly with a CIN 2 or worse endpoint, and the Hosmer Lemeshow test confirmed this correlation to be 92%.

Interpreting colposcopic epithelial patterns and subsequently selecting the site for biopsy is a subjective procedure that is strongly correlated with the skill and experience of the colposcopist. 6 We believe that the good predictive performance of colposcopy in our study was influenced by the extensive experience of the colposcopist. The high prevalence of HSIL in the study population may have also contributed to colposcopy performance, as it has been reported that colposcopy is better at detecting more severe lesions. 10

Our findings show that the degree of acetowhite change was the main colposcopic feature that predicted CIN. This was also previously reported by Shaw et al., 30 who found that after evaluating the individual variables of colposcopic findings, the degree of acetowhite change was the only feature significantly associated with cervical intraepithelial neoplasia after multivariate analysis.

## Conclusion

In an ASC-H population, there is a high rate of severe underlying disease, and colposcopy performed by a highly experienced examiner using IFCPC terminology can produce excellent predictive performance to differentiate patients with CIN 1 or less and patients with CIN 2 or worse. Diagnosis of CIN 2 or worse was more frequent in patients with a previous history of cervical dysplasia and pre-menopausal patients. The degree of acetowhite changes was the best colposcopic feature to predict CIN2 or worse.
